# Bibliography

**Published:** 1896-04

**Authors:** 


### Bibliography.

Dental Chemistry and Metallurgy. Fourth Edition; Revised, Enlarged, and with many Illustrations. By Clifford Mitchell, A.M., M.D. The W. T. Keener Company, Chicago, 1896.

This work of dental chemistry was originally reviewed in this journal upon the issuing of the first edition, with the feeling, after careful examination, that no more thorough text-book on chemistry had up to that time been published for the use of dental students, and this is more than ever emphasized on an examination of this, the fourth edition.

The author says in his preface, "The present edition of the 'Dental Chemistry' is an effort to provide the student with a course which shall include a large number of exercises in experimental chemistry, both inorganic and organic. More than one hundred new pages have therefore been inserted in the book, which are entirely practical in character. . . . Greater attention is also paid in this edition to the germ theory, ptomaines, leucomaines, and various toxines. . . . Descriptions of various new alkaloids and antiseptics will also be found in the chapters relating to them."

Examination of the new matter added in this edition shows careful condensation and a laudable effort to give all needed information upon topics of importance to the average practitioner, as well as to the undergraduate. If there be an error to be found here, it will probably be discovered to exist in the direction of too great brevity, for in the descriptions of many of the new agents the reader is left with but little more than the name. The difficulty with all writers of text-books is that, in their efforts to keep within reasonable limits, they are led into an unwise condensation at the expense of clearness of statement. This only applies to special parts of this book, and not to it as a whole.

While the author has labored to make this volume a text-book for colleges, he has gradually made it a very important reference-book for dental practitioners. In this direction former editions have been found of special value to the writer, and will be increasingly so in this the fourth. It certainly deserves a wide circulation among those who desire to be intelligent upon chemical matters.

Health Notes for Young Wives. By Aimée Raymond Schroeder, M.D. William Wood & Company, New York, 1895.

This book is intended for the instruction of young mothers during pregnancy and subsequent to the birth of the child.

The anxiety of this period can be measurably lessened by an intelligent comprehension of what is required on the part of the young mother. This the author of the small volume succeeds admirably in giving in a clear and satisfactory manner. The information upon its pages, if read, should add to the intelligence of every woman passing through this trying period.

While the writer states that the description of diseases is "foreign to this work," it would have increased materially its value if an additional chapter had been added on the care of infants previous to and during dentition.

It is one of the great errors in the education of young girls in that they are not taught much of the wisdom contained in this book before marriage, but if this has been neglected, this volume will become an excellent educator subsequently.

Catching's Compendium of Practical Dentistry for 1895. B. H. Catching, D.D.S., Editor and Publisher, Atlanta, Georgia, 1896.

The issue of this important review of the labors of 1895 in dentistry comes to us promptly in March. The editor announces that he has added a new department, that of the "Science of Dentistry."

This volume contains abstracts from all the writings of the year worthy of special mention, or which may contain anything of a novel or of a valuable character.

Whenever this compendium is received it gives the impression of great labor involved in its preparation. If the articles could be copied into type exactly as originally printed the task would be comparatively an easy one, but were this plan adopted the volume would be bulky, and the readers would be few and far between. Hence condensation is important, indeed necessary.

It is strange that this effort of Dr. Catching has not met with the financial success its merits deserve. It is hard to understand this, for if there is one thing the dental practitioner needs it is to have the literature of the year in dentistry brought to him in the most concise form, and to be of easy reference. There is not a man of the fifteen thousand dentists in the country who can afford to neglect any opportunity to keep in touch with the everchanging panorama of prosthetic, operative, and scientific dentistry.

Many, doubtless, have felt and will continue to feel that there is not much of novelty as we go on year by year. This may be true, but the few grains of wheat to the bushel of chaff must be garnered that they may serve a purpose in the nourishment of the entire professional body. No calling can do without a *résumé* of its work year by year, and the three dollars necessary to secure this book should be forthcoming from a wide circle to encourage the publisher to continue this work upon ever extending lines.

It is gratifying to notice that the editor has added a department devoted to the science of dentistry. This greatly increases the value of the compendium, and will no doubt, in the future, be of ever-growing interest.

The publisher has been obliged to increase the price to three dollars. This slight increase is fully warranted by the heavy expense attendant upon the collection of material both at home and abroad, and should not prove a serious obstacle to its increased circulation.

